# Single zoledronic acid infusion as a cause of acute kidney impairment requiring dialysis in two patients with osteoporosis

**DOI:** 10.20945/2359-4292-2024-0159

**Published:** 2024-11-06

**Authors:** Djordje Marina, Charlotte Ejersted, Kristine Hommel, Peter Schwarz

**Affiliations:** 1 Department of Endocrinology Copenhagen Denmark Department of Endocrinology, Rigshospitalet, Copenhagen, Denmark; 2 Odense University Hospital Department of Endocrinology Odense Denmark Department of Endocrinology, Odense University Hospital, Odense, Denmark; 3 Holbæk Hospital Department of Nephrology Holbæk Denmark Department of Nephrology, Holbæk Hospital, Holbæk, Denmark; 4 University of Copenhagen Faculty of Health Sciences Copenhagen Denmark Faculty of Health Sciences, University of Copenhagen, Copenhagen, Denmark

## Abstract

Zoledronic acid is a widely used bisphosphonate for treating osteoporosis and hypercalcemia related to malignancy. It is also used to prevent bone loss induced by cancer treatment and bone metastases in various cancer types. Zoledronic acid is safe for most patients and is generally not associated with severe side effects. However, there have been reports of acute kidney impairment occurring after administration of intravenous zoledronic acid, mostly in patients with cancer (who received a high cumulative dose of this medication) or preexisting kidney impairment, and in patients with a history of nephrotoxic treatment. We report herein the cases of two patients without history of cancer, who developed dialysis-requiring acute kidney impairment after a single administration of intravenous zoledronic acid. None of the patients had previously used nephrotoxic medications, and one of them had a normal kidney function before zoledronic acid treatment. To the best of our knowledge, this report describes the first case of acute kidney impairment in a patient without risk factors. The findings of this report show that acute kidney impairment following intravenous zoledronic acid treatment can also occur in low-risk patients, highlighting the need for monitoring kidney function in all patients receiving this treatment.

## INTRODUCTION

The bisphosphonate zoledronic acid is widely used to treat osteoporosis, malignancy-related hypercalcemia, and Paget's disease (1). Bisphosphonates are also used as a treatment to reduce breast cancer recurrence rates in bone and improve breast cancer survival in postmenopausal women with estrogen-receptor-positive disease treated with aromatase inhibitors (2). Due to their powerful effect on preventing bone loss, bisphosphonates are also widely and frequently examined in clinical trials with preselected patients who may not have various comorbidities. Although the favorable safety profile of bisphosphonates has been extensively documented, serious adverse events, including kidney toxicity, have been reported (3). The occurrence of acute kidney impairment is most often reported in patients with cancer receiving high cumulative doses of intravenous zoledronic acid. The potential renal effects of bisphosphonates are particularly important because these agents are primarily excreted through the kidneys, and patients undergoing bisphosphonate treatment often have various underlying health conditions.

We report herein two cases of acute kidney impairment following a single dose of intravenous zoledronic acid for the treatment of osteoporosis, one in a patient with no previous kidney disease and the other in a patient with previous chronic kidney failure. Both patients had not been previously diagnosed with cancer or used nephrotoxic medications.

## CASE REPORT #1

A 77-year-old female patient was referred to the Endocrinology Outpatient Clinic for evaluation of osteoporosis due to vertebral fractures and severe back pain. The patient's medical history included a diagnosis of seropositive rheumatoid arthritis (at the age of 30 years), hypertension, hypercholesterolemia, atrial flutter, moderate aortic stenosis, acute myocardial infarction, and percutaneous coronary intervention (at the age of 52 years). Due to her diagnosis of rheumatoid arthritis, the patient had been treated with immunosuppressant leflunomide and injections of betamethasone. Prednisolone 10 mg/day was added during the previous 5 years for symptom control. Because of side effects, the patient was periodically treated with methotrexate, infliximab, and sulfasalazine, although she did not use these medications during the year prior to her referral.

An initial bone evaluation using dual-energy X-ray absorptiometry (DXA) scanning showed T-scores of +1.1 in the spine, −0.4 in the total hip, and −0.9 in the femoral neck. An X-ray evaluation showed vertebral compression fractures of 25% in T9 and T12. On biochemical analysis, ionized calcium, 25-hydroxyvitamin D, parathyroid hormone (PTH), electrolytes, liver and kidney function parameters were all within the reference range (RR), including creatinine (78 µmol/L [RR 45-90 µmol/L]) and estimated glomerular filtration rate (eGFR; 66 mL/min/1.73 m^2^ [RR > 60 mL/min/1.73 m^2^]). Measurement of myeloma protein was negative. Since the patient's medical history was negative for cancer, treatment with recombinant parathyroid hormone analog was offered, but the patient declined it due to its cost and requirement for daily subcutaneous injections. Treatment with an annual 5 mg dose of intravenous zoledronic acid was then recommended and initiated after agreed by the patient.

Zoledronic acid was administered via a 15-minute infusion. One week after the infusion, the patient was admitted to the emergency department with flu-like symptoms and aggravation of back pain. A biochemical evaluation showed acute kidney impairment (creatinine 170 µmol/L, eGFR 25 mL/min/1.73 m^2^, and urea 8.5 mmol/L [RR 3.1-7.9 mmol/L]), a sign of infection (C-reactive protein level of 180 mg/L [RR < 8 mg/L] but normal leukocyte count), slight hypoalbuminemia (albumin level of 33 g/L [RR 34-45 g/L]), hyponatremia (sodium level of 133 mmol/L [RR 137-145 mmol/L]), and low hemoglobin level (6.5 mmol/L [RR 7.3-9.5 mmol/L]). Due to kidney function impairment, rosuvastatin, ezetimibe, and losartan were discontinued. A urine strip test was positive for erythrocytes and leucocytes and negative for nitrates, and analysis of a 24-hour urine specimen showed proteinuria (1.0 g/day [RR < 0.3 g/day]). Both urine and blood cultures were negative. A computed tomography scan of the abdomen, obtained without intravenous contrast, showed slight bilateral perirenal stranding, without hydronephrosis or other changes in the intra-abdominal organs.

**Figure 1:**
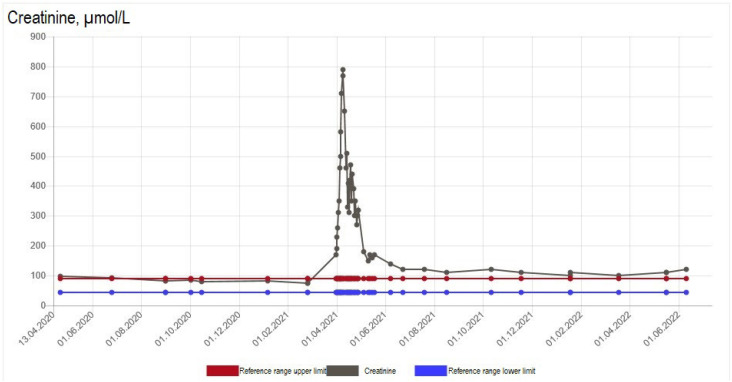
Serum creatinine levels over 1 year before and after the incident of acute kidney impairment in Case #1.

**Figure 2:**
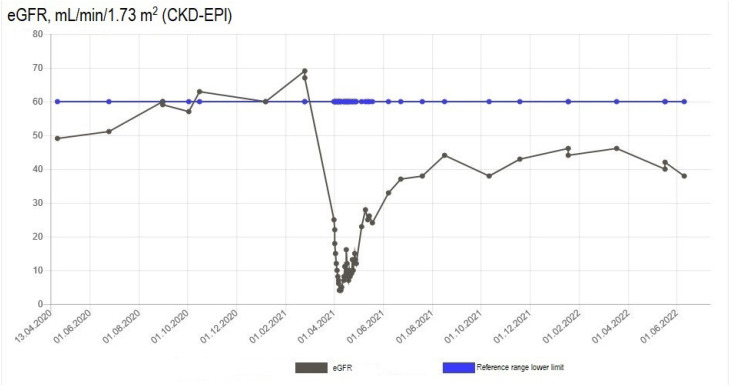
Serum estimated glomerular filtration rate over 1 year before and after the incident of acute kidney impairment in Case #1.

Antibiotics and fluid therapy were administered. Urinary tract infection was suspected, and oral pivmecillinam was initiated. However, due to a further increase in C-reactive protein to 280 mg/L, pivmecillinam was replaced by dose-reduced intravenous piperacillin/tazobactam twice daily and oral clarithromycin. Intravenous fluid administration of 3 L of 0.9% sodium chloride daily was started at admission. Despite fluid therapy, the patient's creatinine level increased on the fourth day (Figure 1), and sepsis-induced acute kidney impairment was suspected. The patient was fitted with an indwelling catheter and maintained a positive fluid balance of 1 L/day. However, 2 days later, her urinary output decreased to 850 mL/day, her kidney function declined further (Figures 1 and 2), and she developed hypoalbuminemia (serum albumin level of 20 g/L).

Two weeks after zoledronic acid infusion, the patient was transferred to the nephrology department and hemodialysis was initiated due to further decline in kidney function, reflected by a creatinine level of 790 µmol/L (Figure 1), eGFR of 4 mL/min/1.73 m^2^ (Figure 2), and urea of 29 mmol/L. Antinuclear antibodies (ANA), antiglomerular basement membrane antibodies (anti-GBM), myeloperoxidase-antineutrophil cytoplasmic autoantibodies (MPO-ANCA), and proteinase 3-antineutrophil cytoplasmic autoantibodies (PR3-ANCA) were negative. High-dose (1 mg/kg) prednisolone was administrated. About 1.5 months later, the patient experienced a recovery in kidney function, leading to discontinuation of hemodialysis. A kidney biopsy showed acute interstitial nephritis, with zoledronic acid suspected as the main cause. Her kidney function stabilized, but the pretreatment level was not achieved 1 year after the acute kidney incident (eGFR 38-45 mL/min/1.73 m^2^) (Figures 1 and 2). The osteoporosis treatment was switched to subcutaneous denosumab 60 mg every 6 months, with no further impact on kidney function observed after 2 months of treatment.

## CASE REPORT #2

A 76-year-old female patient was referred to the Endocrinology Outpatient Clinic due to incidental hypercalcemia. She had a family history of osteoporosis (mother and sister). Her medical history was positive for chronic kidney disease, severe chronic obstructive pulmonary disease, epilepsy, and hypertension, and she had a previous distal radius fracture. Six months before the referral, the patient had a low-energy trauma. Due to low back pain, a radiological examination of the spine was performed and showed a compression fracture in T9, with a 30% height reduction. An initial bone evaluation with DXA scanning was performed and showed T-scores of −2.6 in the spine, −2.3 in the total hip, and −1.7 in the radius.

An initial biochemical evaluation showed levels of ionized calcium of 1.42 mmol/L, PTH of 10.8 pmol/L (RR 1.6-6.0 pmol/L), 25-hydroxyvitamin D of 101 nmol/L (RR 50-160 nmol/L), eGFR of 44 mL/min/1.73 m^2^, creatinine of 106 µmol/L, hyperkalemia (potassium level of 5.1 mmol/L), and hyponatremia (sodium level of 124 mmol/L [RR 137-145 mmol/L]). Due to hypercalcemia secondary to hyperparathyroidism, we investigated mutations in the calcium-sensing receptor and adaptor related protein complex 2 subunit sigma 1 genes, which were negative. A diagnosis of primary hyperparathyroidism was established, and further evaluation with parathyroid scintigraphy before a planned parathyroidectomy was offered, but the patient refused. Measurement of myeloma protein was negative. Osteoporosis treatment with an annual 5 mg dose of intravenous zoledronic acid was then recommended, which the patient accepted.

**Figure 3:**
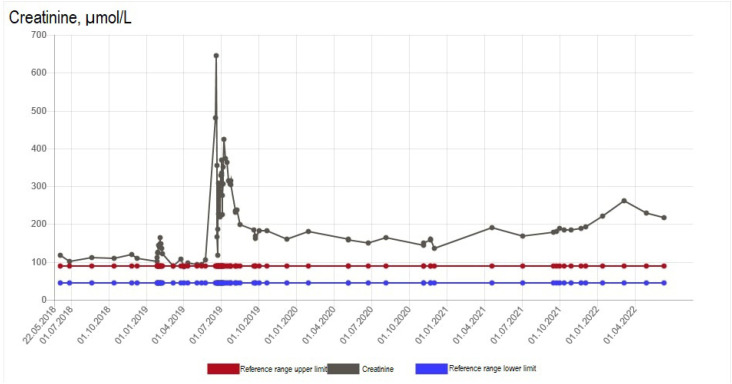
Serum creatinine levels over 1 year before and after the incident of acute kidney impairment in Case #2.

**Figure 4:**
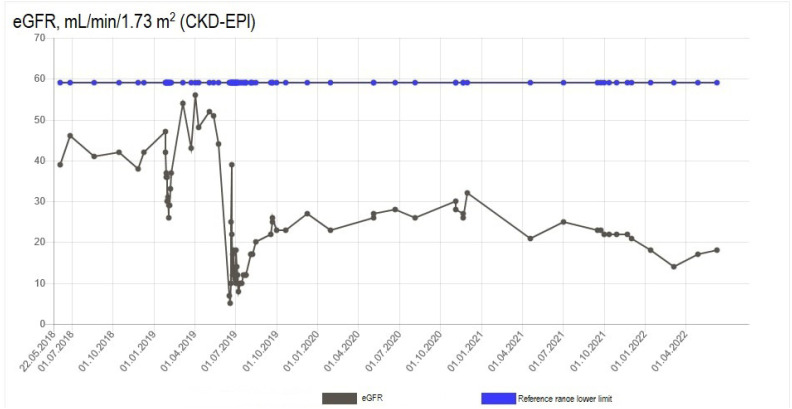
Serum estimated glomerular filtration rate over 1 year before and 3 years after the incident of acute kidney impairment in Case #2.

A few years before the zoledronic acid treatment, the patient had a diagnosis of chronic kidney impairment, with creatinine levels of 100-140 µmol/L (Figure 3) and eGFR of 40-55 mL/min/1.73 m^2^ (RR > 59 mL/min/1.73 m^2^) (Figure 4). Zoledronic acid was administered according to local guidelines as a 30-minute infusion due to eGFR < 45 mL/min/1.73 m^2^. Two weeks after the infusion, the patient was admitted to the emergency department due to malaise, nausea, vomiting, and oliguria, which had progressed over 1 week. At admission, a biochemical evaluation showed an acute exacerbation of her chronic kidney impairment, with levels of creatinine of 645 µmol/L (Figure 3) and urea of 24 pmol/L, metabolic acidosis (pH 7.14, HCO_3_^-^ 11.1 mmol/L, and base excess −16.6 mmol/L), hyperkalemia (potassium level of 5.0 mmol/L), increased C-reactive protein level (48 mg/L [RR < 0.6 mg/L]), and increased leukocyte count (9.62 × 10^9^/L [RR 3.50-8.80 × 10^9^/L]). A blood culture was positive for *Staphylococcus aureus*, and a urine culture was positive for *Proteus vulgaris*. A kidney ultrasound showed a diffuse hyperechoic kidney parenchyma but no hydronephrosis. Slight proteinuria (0.58 g/24h) was recorded (RR < 0.15 g/24h). The patient was not taking any nephrotoxic medication at the time. Autoantibodies (ANA, anti-GBM, MPO-ANCA, and PR3-ANCA) were negative. The patient was admitted, and hemodialysis was started on the first day. Her infection was treated with intravenous cefuroxime and oral ciprofloxacin. After 3 days, the patient resumed spontaneous urination, and the indwelling catheter was discontinued. A transthoracic echocardiography showed no signs of endocarditis.

Two months after the admission, hemodialysis was discontinued due to the patient's regained kidney function. Zoledronic acid was suspected to be the main cause of the acute exacerbation of the patient's chronic kidney impairment, although a kidney biopsy was not performed. During 3 years after the incident, her kidney function remained stable, with creatinine levels of 200-250 µmol/L (Figure 3) and eGFR of 15-20 mL/min 1.73 m^2^ (Figure 4). However, the pretreatment level of kidney function was not fully restored. The patient received no further antiresorptive treatment.

## DISCUSSION

We presented herein two cases of acute kidney impairment requiring dialysis after a single infusion of zoledronic acid for severe osteoporosis. To the best of our knowledge, Case #1 is the first reported case of acute kidney impairment after a single intravenous zoledronic acid treatment in a patient without preexisting neoplasm, kidney impairment, or use of nephrotoxic medications. Her Naranjo probability scale score (4) was +6, indicating that zoledronic acid infusion was the "probable" cause of acute kidney impairment in this patient. A 2016 case report described a patient with known osteoarthritis and a previous diagnosis of squamous cell carcinoma, who was treated with the immunosuppressant tofacitinib, which can increase creatinine levels (5). The patient had a creatinine level within the upper normal RR before receiving a single dose of intravenous zoledronic acid and developed acute kidney impairment histologically verified as being acute tubular nephritis (5). Our patient (Case #1) had acute interstitial nephritis and was also examined for other causes of acute kidney impairment, which were ruled out. Although this patient was hospitalized with flu-like symptoms, these were not likely the cause of her acute kidney impairment, as the patient did not experience nausea, vomiting, or fever.

Our patient in Case #2 had known chronic kidney impairment before her first infusion of zoledronic acid, which is aligned with other similar cases reported in the literature. However, this patient had no history of malignancy or prior use of nephrotoxic medication. Before treatment with zoledronic acid, her creatinine level was elevated, reflecting impaired kidney function. Her Naranjo probability scale score was +5 (4), indicating that intravenous zoledronic acid was the "probable" cause of her acute kidney impairment. Still, *Staphylococcus aureus* sepsis could have contributed to an acute exacerbation of her chronic kidney impairment.

Several cases of kidney impairment after intravenous zoledronic acid have been reported in the literature, but they occurred mostly in patients with a cancer diagnosis or with preexisting kidney impairment. One patient used nephrotoxic medication (nonsteroidal anti-inflammatory drug [NSAID]) and had increased creatinine level/decreased eGFR before intravenous zoledronic acid treatment and onset of acute kidney impairment (6). Another patient had preexisting kidney failure, which had improved spontaneously (7). This patient had a diagnosis of prostate cancer with osteoblastic metastases, and received intravenous zoledronic acid at a dose of 4 mg a month, with a cumulative dose of 116 mg over 29 months before the onset of Fanconi syndrome (7), adding to several other reports of Fanconi syndrome in the literature affecting patients previously diagnosed with cancer (8-10). A published case series of acute kidney impairment after treatment with zoledronic acid included patients with either known chronic kidney impairment or a diagnosis of cancer (11). Another report described the cases of five patients with multiple myeloma and one with Paget's disease who developed acute kidney impairment after an average of 4.7 administrations of monthly intravenous zoledronic acid 4 mg (12). The US Food and Drug Administration published data on 72 patients who developed kidney failure an average of 56 days after receiving zoledronic acid treatment; most of these patients had multiple myeloma or other solid tumors, such as breast or prostate cancer (13). Another publication reported seven patients with different cancer diagnoses who developed acute kidney impairment after zoledronic acid treatment, three of whom with preexisting chronic kidney failure (14). Three of these patients developed permanent kidney damage and one died (14).

Why is intravenous zoledronic acid potentially toxic for the kidneys? About half of this medication is incorporated into bone tissue, and the other half remains in the extracellular fluid (15). Protein-binding of zoledronic acid is very low. Intravenous zoledronic acid bypasses the liver and cytochrome P450 enzyme and is excreted unmetabolized by the kidneys, making its elimination dependent on renal clearance (15). Impaired kidney function can result in reduced excretion of bisphosphonates, leading to their increased levels in bone and serum and resulting in kidney toxicity. Compared with ibandronate, zoledronic acid has a much longer renal half-life (24 days *versus* 150-200 days, respectively) (16), which increases the risk of nephrotoxicity associated with this medication.

Most cases reported on bisphosphonate toxicity relate to toxic acute tubular necrosis. However, many of these reports do not provide results of kidney biopsy or measurement of proteinuria. One of our patients had acute interstitial nephritis on biopsy (Case #1), while the second patient (Case #2) did not undergo this procedure. In general, oral bisphosphonates are associated with a low risk of nephrotoxicity, especially when used for postmenopausal osteoporosis ^3^. The nephrotoxic effect of intravenous zoledronic acid at a dose of 4 mg has been investigated in four osteoporosis trials, and no nephrotoxicity was observed (17-20). However, a transient increase in serum creatinine has been described in some patients (19,20). The main risk factors for nephrotoxicity include advanced cancer, previous exposure to nephrotoxic medicine (NSAID or similar), and type of bisphosphonate (mostly pamidronate) (13). Notably, ibandronate appears to be safe, even in patients with abnormal kidney function at baseline (3).

The level of hydration before zoledronic acid administration requires special attention. Older patients often have difficulties in maintaining normal hydration, which can be a risk factor for the development of kidney insufficiency after administration of zoledronic acid. The most potent bisphosphonates (alendronate, zoledronate, ibandronate, and pamidronate) contain nitrogen, which can inhibit the activity of the enzyme farnesyl diphosphate synthase in bones and kidneys (21), thus blocking the mevalonate pathway which can be the underlying mechanism of renal toxicity (22). Due to these mechanisms and primary renal excretion, clinicians need to have access to a hydration plan for these patients to reduce the risk of nephrotoxicity with zoledronic acid (23). Dai and cols. conducted a retrospective cohort study on older patients, dividing them into four groups based on their fluid plan before and after the administration of zoledronic acid (24). These authors concluded that appropriate hydration prior to zoledronic acid administration is fundamental in reducing the risk of acute kidney impairment (24).

In conclusion, intravenous zoledronic acid may increase the risk of acute kidney impairment. The highest risk of this complication is likely seen in patients with neoplasms (who receive high cumulative doses of the medication, often administered at shorter intervals), in use of nephrotoxic medications (*e.g.*, cytostatic therapies and NSAIDs), or with preexisting kidney impairment. The toxic effect of zoledronic acid seems to be reversible in most, but not in all patients. Our first case involved a patient without neoplasm or use of nephrotoxic medication who developed acute kidney impairment after a single dose of intravenous zoledronic acid. Despite extensive evidence of zoledronic acid use in patients with an eGFR above 35 mL/min/1.73 m^2^, our report suggests that it is important to take an individualized risk approach for patients with osteoporosis who are prescribed intravenous bisphosphonates. It also highlights the importance of ensuring that patients are well-hydrated before infusion. Finally, we suggest prolonging the administration of zoledronic acid to a minimum of 30 minutes for patients with an eGFR of 35-60 mL/min/1.73 m^2^ and, eventually, for older patients with unknown hydration status.

## Data Availability

the patients’ datasets of the research presented in this publication will be made available by the corresponding author upon reasonable request.
